# Psychometric properties of Herth Hope Index in a sample of Portuguese Higher education students using a Rasch model.

**DOI:** 10.1192/j.eurpsy.2026.12006

**Published:** 2026-07-31

**Authors:** C. Laranjeira, A. Querido, M. Dixe

**Affiliations:** 1 School of Health Sciences; 2 ciTechCare, Polytechnic University of Leiria, Leiria, Portugal

## Abstract

**Introduction:**

The emergence of positive psychology has led to an increasing number of scholars investigating the influence of positive psychological attributes on mental well-being. The presence of hope, a significant positive psychological attribute, can mitigate the adverse consequences of psychological instability in secondary school students when they encounter stressful situations.

**Objectives:**

This study aims to assess the HHI´s psychometric characteristics in a sample of Portuguese Higher education students using a Rasch model.

**Methods:**

A methodological study was developed using a sample of Portuguese higher education students enrolled at 5 educational institutions. The snowball e-survey was conducted during spring semester of 2020. Participants completed a personal information sheet and the 12-item HHI, which measures various dimensions of hope.

**Results:**

We received 2227 valid responses during the survey activation period. The mean age of the sample was 22.5±6.2 years (range 18–59 years). Most of the participants were female (79.6%) and 0.9% were diagnosed positive for COVID. After removing these three items (#3, #5, #6), the resulting 9-item scale demonstrated acceptable item fit to the model, acceptable unidimensionality (52.4% of the variance explained), acceptable person goodness-of-fit, adequate separation, and no differential item function. HHI was highly reliable with Rasch-equivalent Cronbach’s alpha statistic of 0.886.

**Conclusions:**

The HHI showed strong one-dimensionality, and the goodness-of-fit values were satisfactory for all the items. The study contributes to conceptualizing hope as a unidimensional construct validated through Rasch analysis while acknowledging the ongoing debate on whether hope in non-clinical contexts is state or trait-oriented.

**Disclosure of Interest:**

None Declared

